# Case Report: Giant communicating solitary fibrous tumor of the temporal skull base and infratemporal fossa

**DOI:** 10.3389/fsurg.2026.1863113

**Published:** 2026-06-22

**Authors:** Changli Han, Zixiao Li, Yang Li, Xiaochuan Guo, Zhipeng Li, Zihao Jiang, Shuangyue Shen, Zhang Xiong

**Affiliations:** Department of Neurosurgery, The First Affiliated Hospital of Wannan Medical College, Yijishan Hospital of Wannan Medical College, Wuhu, China

**Keywords:** case report, craniofacial approach, infratemporal fossa, skull base, solitary fibrous tumor, STAT6

## Abstract

**Background:**

Solitary fibrous tumor (SFT) is a rare mesenchymal spindle-cell neoplasm that can arise in the central nervous system and the head and neck region. Giant communicating lesions spanning the temporal skull base and infratemporal fossa are exceptionally uncommon and remain diagnostically and surgically challenging.

**Case presentation:**

We report a 76-year-old man who presented with more than 3 years of intermittent headache and dizziness, recent symptom progression, and a palpable right maxillofacial mass. Magnetic resonance imaging demonstrated a strongly enhancing right temporal skull base lesion extending through the foramen ovale into the infratemporal/masticator space, measuring approximately 74 × 61 mm. Preoperative MR angiography did not show an obvious close relationship between the tumor and adjacent major vessels. After multidisciplinary evaluation, the patient underwent combined craniofacial tumor resection and skull base reconstruction performed jointly by neurosurgery and oral and maxillofacial surgery. Histopathology showed a spindle-cell neoplasm with alternating cellularity and staghorn-like vessels, 1–2 mitotic figures per 10 high-power fields, no necrosis, and no marked cytologic atypia. Immunohistochemistry showed nuclear STAT6 positivity together with CD34, CD99, and Bcl-2 expression and a Ki-67 labeling index of approximately 5%, supporting the diagnosis of central nervous system World Health Organization grade 1 SFT. The postoperative course was uneventful, symptoms improved, and no recurrence was reported on outside follow-up magnetic resonance imaging at 22 months.

**Conclusion:**

This case adds to the limited literature on skull base SFT with intracranial-extracranial extension and highlights the importance of careful clinicoradiological assessment, multidisciplinary maximal safe resection, pathological confirmation with STAT6, and sustained radiological surveillance.

## Introduction

Solitary fibrous tumor (SFT) is a rare fibroblastic mesenchymal neoplasm originally described in the pleura but now recognized across a broad range of extrapleural sites. Involvement of the head and neck is uncommon, whereas lesions centered at the skull base or infratemporal fossa are particularly rare. Because of their deep location, potential vascularity, and proximity to critical neurovascular structures, these tumors often pose substantial diagnostic and operative challenges (1–3).

At present, definitive diagnosis relies on histomorphology and immunohistochemistry, with nuclear STAT6 expression serving as a robust surrogate marker for the NAB2::STAT6 fusion that characterizes SFT (4–6). Radiologically, however, skull base SFT frequently overlaps with trigeminal schwannoma, meningioma, and other spindle-cell tumors, making preoperative diagnosis difficult. Here, we report a giant communicating SFT involving the right temporal skull base and infratemporal fossa and highlight the imaging clues, multidisciplinary surgical strategy, reconstructive considerations, and follow-up implications relevant to clinical practice.

## Case description

A 76-year-old man presented with intermittent headache and dizziness for more than 3 years, with clear worsening during the month before admission. He also noticed a gradually palpable mass in the right maxillofacial region. The patient had no documented history of major systemic comorbidity, previous craniofacial surgery, previous radiotherapy, or long-term regular medication use. He denied relevant smoking or alcohol history. On admission, he was conscious and cooperative. Physical examination revealed a palpable right maxillofacial mass. Neurological and craniofacial examination did not show limb motor or sensory deficit, facial sensory loss, facial weakness, dysarthria, or lower cranial nerve dysfunction. No clinically apparent impairment of mastication or jaw opening was noted. Oral cavity examination did not reveal an intraoral mass, mucosal ulceration, or tooth-related infection. The patient denied diplopia, visual deterioration, orbital pain, hearing loss, tinnitus, otalgia, and otorrhea. Because the symptoms had progressed and the lesion appeared surgically approachable on imaging, further multidisciplinary evaluation was undertaken.

Preoperative magnetic resonance imaging (MRI) demonstrated a large mass involving the right temporal region and skull base, with transcranial extension along the foramen ovale into the extracranial compartment. The lesion was centered at the right middle cranial fossa floor/temporal skull base, extended through the foramen ovale in close proximity to the mandibular division of the trigeminal nerve (V3), and occupied the deep infratemporal/masticator space. The medial margin of the lesion was adjacent to the middle cranial fossa floor, but the available MRI showed no definite internal carotid artery encasement or gross cavernous sinus invasion. Preoperative MR angiography (MRA) was also reviewed and did not show an obvious close relationship between the tumor and adjacent major vessels, including the internal carotid artery. The lesion was isointense to slightly hypointense on T1-weighted imaging, showed mixed iso- to slightly hypointense signal on T2-weighted and fat-suppressed T2-weighted imaging, and displayed mixed high and low signal intensity on diffusion-weighted imaging. After gadolinium administration, the lesion showed marked heterogeneous enhancement. The maximal cross-sectional diameter was approximately 74 × 61 mm. Adjacent dural thickening and enhancement were also noted. In addition, a patchy abnormal signal was identified in the right temporal lobe, appearing hypointense on T1-weighted imaging, hyperintense on T2-weighted imaging, and hypointense on T1-FLAIR and diffusion-weighted imaging; this area appeared to be locally connected to the posterior horn of the lateral ventricle. CT, CT angiography, digital subtraction angiography, and preoperative embolization were not performed. Because MRA did not suggest obvious major-vessel encasement or close major-vessel involvement, and because the planned combined craniofacial approach allowed direct exposure and intraoperative vascular control, additional angiographic evaluation or embolization was not pursued. Although the imaging findings strongly suggested a skull base neoplasm, they were not specific enough to reliably distinguish SFT from more common entities such as trigeminal schwannoma or meningioma (Figure 1).

**Figure 1:**
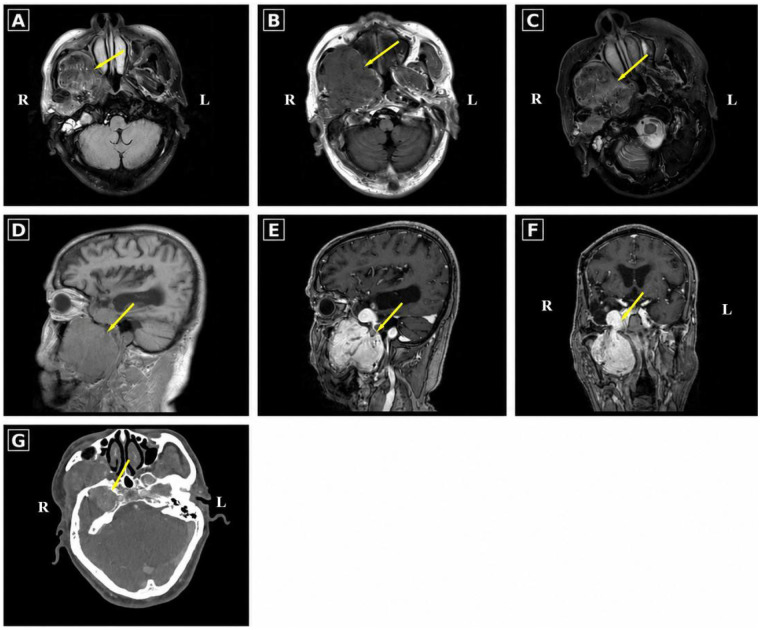
Preoperative magnetic resonance imaging and MR angiography. Axial images **(A–C)**, sagittal images, **(D,E)**, and coronal image **(F)** show a large right temporal skull base mass extending through the foramen ovale into the infratemporal fossa. **(A)** Axial T2WI-FLAIR, **(B)** axial T1WI, **(C)** axial T2WI, **(D)** sagittal T1WI, **(E)** sagittal contrast-enhanced T1WI, and **(F)** coronal contrast-enhanced T1WI. Post-contrast images reveal marked heterogeneous enhancement of the lesion. **(G)** Preoperative MR angiography shows no obvious close relationship between the tumor and adjacent major vessels.

## Diagnostic assessment

The principal preoperative differential diagnoses included trigeminal schwannoma, skull base meningioma, hemangiopericytoma/SFT, and other spindle-cell or vascular tumors. The lesion's transforaminal intracranial-extracranial growth pattern, relationship to the foramen ovale/V3 pathway, and avid contrast enhancement were compatible with several of these possibilities, and no single imaging sign was pathognomonic. Therefore, the preoperative strategy emphasized complete multidisciplinary planning rather than reliance on radiology alone.

Histologically, the tumor was composed of spindle cells arranged in alternating hypocellular and hypercellular areas. Prominent thin-walled branching vessels with a staghorn-like configuration were identified. The mitotic count was low, with approximately 1–2 mitotic figures per 10 high-power fields. No definite tumor necrosis, marked cytologic atypia, or adipocytic component was seen in the examined sections. Immunohistochemistry showed nuclear positivity for STAT6 and positive staining for CD34, Bcl-2, and CD99, whereas S-100 and GFAP were negative; the Ki-67 labeling index was approximately 5% (Figure 2). Taken together with the characteristic morphology, low mitotic activity, absence of necrosis or marked atypia, and STAT6 immunopositivity, these findings supported the diagnosis of SFT, central nervous system World Health Organization grade 1. NAB2::STAT6 fusion testing was not performed; therefore, nuclear STAT6 immunohistochemistry was used as a surrogate diagnostic marker, and the lack of molecular confirmation was considered a limitation.

**Figure 2:**
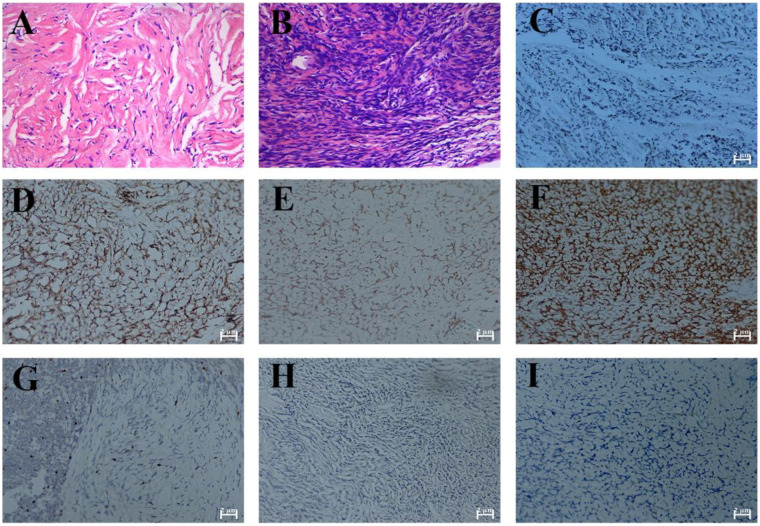
Histopathological and immunohistochemical findings (100 ×). H&E shows storiform spindle cells and staghorn-like vessels **(A,B)**. Immunohistochemistry shows nuclear STAT6 positivity and positive staining for CD34, Bcl-2, and CD99 **(C–F)**. Ki-67 shows a proliferation index of approximately 5% **(G)**, whereas S-100 **(H)** and GFAP **(I)** are negative.

## Therapeutic intervention and follow-up

After multidisciplinary discussion, the patient underwent combined resection of the communicating craniofacial tumor and skull base reconstruction under general anesthesia. The patient was placed supine with a shoulder roll, the head rotated approximately 60 degrees to the left, and the head secured in a fixation frame. The planned right frontotemporal scalp incision, right cervicofacial incision, and an approximately 10 cm longitudinal incision over the lateral aspect of the right thigh for graft harvest were marked before routine sterile preparation and draping. The oral and maxillofacial surgery team first performed the extracranial exposure. A cervicofacial incision was made from the right oral commissure inferiorly and posteriorly along a line approximately 2.0 cm below the mandibular border to the submastoid region. The skin, subcutaneous tissue, and platysma were divided in layers, the flap was elevated in the deep plane of the platysma, and the inferior mandibular border was exposed. The marginal mandibular branch of the facial nerve was carefully identified and protected, and the facial artery and vein were ligated. An additional intraoral vestibular incision was then made in the right upper and lower buccal sulcus, allowing elevation of the right facial soft tissues and exposure of the right maxilla and mandibular ramus.

To improve access to the deep tumor bed, the coronoid process and anterior margin of the right mandibular ramus were resected, after which the posteriorly situated mass was identified. Sharp and blunt dissection was carried out along the tumor margin from the maxillofacial side toward the skull base to mobilize the extracranial component and facilitate subsequent intracranial resection. The neurosurgical team then re-prepared the field and performed a right frontotemporal craniotomy with zygomatic exposure. The scalp was incised to the pericranium, the temporalis muscle and superficial temporal fascia were dissected, and the zygomatic arch and frontotemporal bone were exposed. After temporary removal of the zygomatic arch, two burr holes were placed and a bone flap was fashioned. The sphenoid ridge was drilled down to flatten the skull base and optimize exposure of the middle cranial fossa floor. Following circumferential dural suspension, the dura was opened in a curvilinear fashion and the lesion was approached along the temporal skull base. Intraoperatively, the tumor was gray-yellow, firm, and highly vascular, with a broad attachment to the temporal skull base and extension through a destructive skull base defect into the maxillofacial compartment. Internal debulking was performed first to reduce tumor volume, followed by stepwise division of the skull base attachment and meticulous circumferential dissection of the intracranial and extracranial components under direct visualization. This combined sequence allowed piecemeal removal of the communicating craniofacial lesion from both corridors.

After tumor removal, the intracranial and maxillofacial operative fields were carefully inspected. No gross residual tumor was identified by the operating teams, and gross total resection was therefore judged intraoperatively. Because the lesion was removed piecemeal, the reported specimen measuring approximately 3.0 × 3.0 × 2.5 cm represented the portion of tumor tissue submitted to the Department of Pathology for diagnostic evaluation rather than the aggregate tumor size. The remaining tumor fragments were transferred, with consent from the patient's family, to the tumor specimen bank of our hospital. The total aggregate specimen size and weight were not reliably recorded, whereas the preoperative imaging measurement of 74 × 61 mm better reflected the overall lesion extent. Meticulous hemostasis was achieved using bipolar coagulation and adjunctive hemostatic materials. Autologous fat and fascia lata harvested from the right thigh were used for multilayer skull base reconstruction. Instrument and sponge counts were correct, the dura was closed in a watertight fashion, and the bone flap was replaced and secured with fixation plates. The oral and maxillofacial team then reinspected the deep skull base-maxillofacial component and confirmed satisfactory hemostasis. A right cervical negative-pressure drain was placed, the temporalis muscle and surrounding soft tissues were reapproximated, the zygomatic arch was restored, and the cranial and cervicofacial incisions were closed in layers with pressure dressing applied. The anesthetic course remained stable throughout the procedure. The patient received 2 units of suspended red blood cells and 400 mL of plasma intraoperatively without transfusion reaction. Representative intraoperative photographs are shown in Figure 3.

**Figure 3:**
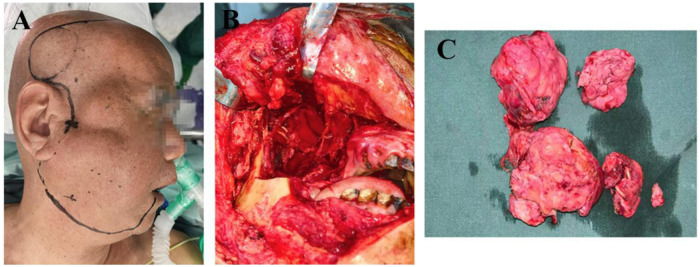
Representative intraoperative photographs of the combined craniofacial approach and tumor resection. **(A)** Preoperative surface marking showing the planned right frontotemporal scalp incision and cervicofacial incision; the patient has been de-identified. **(B)** Transfacial operative exposure of the deep maxillofacial/skull base compartment during dissection of the extracranial component of the communicating tumor. **(C)** Resected tumor specimens after piecemeal removal of the communicating lesion.

Postoperatively, the patient was admitted to the intensive care unit with tracheal intubation. By postoperative day 3, his vital signs were stable, consciousness was clear, and he was transferred to the general ward. Supportive treatment included infection prophylaxis, fluid replacement, nutritional support, and antiepileptic therapy. No adjuvant radiotherapy or chemotherapy was administered. On postoperative day 21, headache and dizziness had improved compared with the preoperative state. No cerebrospinal fluid leak, wound infection, seizure, new facial weakness, facial sensory deficit, or clinically apparent deterioration in mastication or jaw opening was documented, and the patient was discharged. Because the patient lived far from our institution, he was advised to undergo scheduled MRI surveillance at a local hospital, and telephone follow-up was conducted by our team. Immediate postoperative MRI was not available in our institutional record; therefore, the extent of resection was assessed intraoperatively and by subsequent local surveillance imaging. At 22-month telephone follow-up, headache and dizziness remained markedly improved, no delayed postoperative complications were reported, and the outside-institution MRI report showed no evidence of recurrence. Based on the postoperative plan, contrast-enhanced MRI covering the brain/skull base and infratemporal-maxillofacial operative field was recommended every 6 months during the first 2 years, annually during years 3–5, and every 1–2 years thereafter as lifelong surveillance, with earlier imaging if new symptoms occurred. The clinical timeline of the episode of care is summarized in Table 1.

**Table 1:** Timeline of the episode of care.

Time point	Clinical course
> 3 years before admission	Intermittent headache and dizziness began.
1 month before admission	Symptoms worsened and the right maxillofacial mass became clinically evident.
Admission	The patient had no documented major systemic comorbidity, previous craniofacial surgery, previous radiotherapy, or long-term regular medication use, and denied relevant smoking or alcohol history. Physical examination showed a right maxillofacial mass without limb motor/sensory deficit, facial weakness, facial sensory loss, lower cranial nerve dysfunction, mastication difficulty, jaw-opening limitation, oral cavity lesion, ophthalmologic complaint, or otologic complaint.
Preoperative imaging	MRI showed a markedly enhancing right temporal skull base lesion extending through the foramen ovale into the infratemporal/masticator space, approximately 74 × 61 mm. The lesion was related to the foramen ovale/V3 corridor and middle cranial fossa floor. MRA did not show an obvious close relationship between the tumor and adjacent major vessels, including the internal carotid artery. CT/CTA/DSA and preoperative embolization were not performed.
Index surgery	Combined craniofacial tumor resection and skull base reconstruction were performed jointly by neurosurgery and oral and maxillofacial surgery under intraoperative neurophysiological monitoring. The tumor was removed piecemeal from both corridors, and gross total resection was judged intraoperatively after inspection of the intracranial and maxillofacial operative fields. The 3.0 × 3.0 × 2.5 cm tissue size referred to the portion submitted for pathology; remaining fragments were transferred to the institutional tumor specimen bank with family consent.
Postoperative day 3	Vital signs were stable, consciousness was clear, and the patient was transferred from the intensive care unit to the general ward.
Postoperative day 21	Headache and dizziness improved. No cerebrospinal fluid leak, wound infection, seizure, new facial weakness, facial sensory deficit, or obvious mastication/jaw-opening deterioration was documented. The patient was discharged without adjuvant radiotherapy or chemotherapy.
22-month follow-up	At 22 months, symptoms remained improved and outside MRI showed no evidence of recurrence. We recommended contrast-enhanced MRI of the brain/skull base and operative field every 6 months in years 0–2, annually in years 3–5, and then every 1–2 years lifelong; earlier imaging if symptoms recur.

## Discussion

This case represents an uncommon presentation of SFT as a giant communicating skull base tumor extending from the right temporal region into the infratemporal fossa through the foramen ovale. Although SFT is increasingly recognized across multiple anatomic sites, oral and maxillofacial involvement remains rare, and lesions crossing the skull base into the infratemporal compartment are particularly unusual. From a clinical perspective, such tumors are important because their size, deep location, and transforaminal growth pattern can obscure the diagnosis and complicate surgical planning.

Previously published infratemporal fossa or skull base SFT cases have emphasized a similar diagnostic dilemma: tumors may show avid enhancement and well-defined margins but still mimic schwannoma, meningioma, paraganglioma, or other vascular/spindle-cell tumors before pathology (3, 7, 8). In comparison with smaller or more localized infratemporal lesions, the present case was notable for its large size, middle cranial fossa involvement, extension through the foramen ovale along the expected V3 corridor, and need for a combined craniofacial approach. These features make the case clinically instructive rather than merely descriptive.

In this patient, MRI demonstrated a strongly enhancing lesion with marked intracranial-extracranial extension, but the imaging phenotype was not disease-specific. The relationship to the foramen ovale and masticator space supported inclusion of trigeminal schwannoma in the differential diagnosis, whereas the dural-based component and avid enhancement also raised the possibility of meningioma. Preoperative MRA did not show an obvious close relationship between the tumor and adjacent major vessels. Nevertheless, preoperative CT could have better characterized skull base bone destruction, and CTA or DSA could have provided more detailed information about tumor vascular supply and the feasibility of embolization. These additional studies were not obtained in this case, and this should be considered when interpreting the operative planning. In practice, the final diagnosis still depended on postoperative pathological assessment. In the present case, the combination of spindle-cell morphology, alternating cellularity, branching thin-walled vessels, and nuclear STAT6 positivity provided persuasive evidence supporting SFT (5, 6, 9).

A major feature of this case was the bidirectional growth of the tumor across the skull base. A single operative corridor would probably not have allowed sufficient visualization of both the intracranial and infratemporal components or safe control of the skull base attachment. For this reason, a combined craniofacial strategy involving neurosurgery and oral and maxillofacial surgery was selected. This approach enabled coordinated exposure from above and below, stepwise internal decompression, circumferential dissection, and subsequent reconstruction of the skull base defect with autologous fat and fascia lata. Our experience is consistent with prior head and neck SFT reports showing that tailored multi-corridor approaches may be necessary to maximize resection while preserving adjacent neurovascular structures (7, 10, 11).

Another clinically relevant issue is postoperative management, particularly whether adjuvant radiotherapy should be given after gross total resection of a low-grade CNS SFT. Complete surgical resection remains the central treatment goal for CNS SFT, and postoperative radiotherapy is more consistently considered for subtotal resection, recurrent disease, high-grade tumors, or residual/positive-margin disease. However, for a completely resected World Health Organization grade 1 lesion, the benefit-risk balance remains less clearly defined because most available evidence is retrospective and combines tumors of different grades and extents of resection (12–14). A recent systematic review and meta-analysis focusing on intracranial SFT after gross total resection reported improved progression-free and overall survival in pooled cohorts receiving postoperative radiotherapy, but grade-specific evidence for fully resected grade 1 disease remains limited and heterogeneous (15). In the present patient, the tumor showed low mitotic activity, absence of necrosis or marked cytologic atypia, a Ki-67 labeling index of approximately 5%, and intraoperative gross total resection. Therefore, adjuvant radiotherapy was withheld after multidisciplinary consideration, and radiotherapy would be reconsidered if postoperative imaging showed residual disease, progression, or recurrence.

Although adjuvant treatment was not administered, surveillance should not be relaxed. CNS SFT can recur or metastasize after prolonged intervals, and low histological grade does not completely eliminate later risk (16–18). We therefore recommended contrast-enhanced MRI of the brain/skull base and infratemporal-maxillofacial operative field every 6 months during the first 2 years, annually during years 3–5, and every 1–2 years thereafter as lifelong surveillance, with immediate imaging if new headache, cranial nerve symptoms, jaw-opening or masticatory difficulty, or recurrent maxillofacial swelling occurs. Periodic systemic imaging, such as chest-abdomen CT or whole-body PET/CT, may also be considered during long-term follow-up because extracranial metastasis has been reported, although optimal systemic surveillance intervals remain undefined (17, 18).

This report has several limitations. First, it describes a single patient, and the 22-month follow-up remains relatively short for SFT, a tumor type that may recur late. Second, NAB2::STAT6 fusion testing was not performed; therefore, the molecular diagnosis relied on STAT6 immunohistochemistry as a surrogate marker. Third, although MRA did not show an obvious close relationship between the tumor and adjacent major vessels, preoperative CT, CTA, DSA, and embolization were not performed, limiting the assessment of skull base bone destruction and tumor vascular supply. Fourth, immediate postoperative MRI was not available in the institutional record, and the 22-month MRI was obtained at an outside institution after local surveillance because the patient lived far from our hospital; therefore, postoperative and follow-up imaging figures could not be displayed. Finally, some craniofacial functional outcomes, such as formal jaw-opening distance measurement and formal ophthalmologic or otologic testing, were not collected prospectively. These limitations should be considered when interpreting the conclusions.

Overall, this case adds to the limited clinical literature on communicating skull base-infratemporal fossa SFT and provides several practical implications. First, a markedly enhancing skull base mass with transforaminal intracranial-extracranial extension should prompt consideration of SFT in the differential diagnosis. Second, definitive classification still relies on pathological assessment, with STAT6 serving as a particularly informative marker. Third, multidisciplinary maximal safe resection combined with skull base reconstruction can result in favorable short- to mid-term outcomes in carefully selected patients. Additional case accumulation and longer follow-up are needed to clarify recurrence patterns and refine management strategies for these rare lesions.

Finally, the educational value of the present case is also supported by the observations of Gubian et al., who emphasized that histopathology combined with immunohistochemistry, particularly STAT6 together with markers such as CD34, Bcl-2, and vimentin, is central to the diagnosis and management of intracranial SFTs. Our case is concordant with this principle because the diagnosis was established only after integrating classic spindle-cell morphology, staghorn-like vasculature, low mitotic activity, and nuclear STAT6 with CD34 and Bcl-2 expression. Although low-grade SFTs are often clinically indolent and our patient remained recurrence-free at 22 months, Gubian et al. also highlighted the uncertain and sometimes unpredictable behavior of intracranial SFTs. Therefore, even apparently benign or World Health Organization grade 1 lesions should be managed with careful pathological confirmation, individualized postoperative decision-making, and prolonged radiological surveillance (19). Furthermore, Baněčková et al. revisited solitary fibrous tumors of the head and neck region and emphasized that these rare tumors require integrated clinicopathological assessment and careful long-term follow-up (20).

## Patient perspective

The patient reported clear improvement in headache and dizziness after surgery and was satisfied with the treatment result. He agreed with continued long-term MRI follow-up because of the possibility of delayed recurrence.

## Data Availability

The original contributions presented in the study are included in the article/Supplementary Material, further inquiries can be directed to the corresponding author.

## References

[1] Stanisce L Ahmad N Levin K Deckard N Enriquez M Brody J. Solitary fibrous tumors in the head and neck: comprehensive review and analysis. Head Neck Pathol. (2020) 14:516–24. 10.1007/s12105-019-01058-6 31338745 PMC7235103

[2] Wang C Wang B He J. Solitary fibrous tumors of the oral and maxillofacial region: a case series from a single-center. BMC Oral Health. (2024) 24:1444. 10.1186/s12903-024-05241-2 39604942 PMC11603982

[3] Nini K Aziz Z Ndelafei D Ghazoui H Hattab MSK Hattab NM. Solitary fibrous tumors of the infratemporal fossa: case series. J Surg Case Rep. (2025) 2025(5):rjaf338. 10.1093/jscr/rjaf338 40417050 PMC12100735

[4] Louis DN Perry A Wesseling P Brat DJ Cree IA Figarella-Branger D. The 2021 WHO classification of tumors of the central nervous system: a summary. Neuro-Oncology. (2021) 23:1231–51. 10.1093/neuonc/noab106 34185076 PMC8328013

[5] Thway K Ng W Noujaim J Jones RL Fisher C. The current status of solitary fibrous tumor: diagnostic features, variants, and genetics. Int J Surg Pathol. (2016) 24:281–92. 10.1177/1066896915627485 26811389

[6] Ren C D'Amato G Hornicek FJ Tao H Duan Z. Advances in the molecular biology of the solitary fibrous tumor and potential impact on clinical applications. Cancer Metastasis Rev. (2024) 43:1337–52. 10.1007/s10555-024-10204-8 39120790 PMC11554739

[7] Naresh KP Parida Pradipta K Mahesha V Das A. Solitary fibrous tumor of the infratemporal fossa: a case report. Indian J Otolaryngol Head Neck Surg. (2007) 59:145–7. 10.1007/s12070-007-0042-1 23120414 PMC3451794

[8] Freiser ME Castaño JE Whittington EE Arnold DJ Sidani CA. Solitary fibrous tumor of the infratemporal fossa. J Radiol Case Rep. (2014) 8(12):1–8. 10.3941/jrcr.v8i12.1742 25926911 PMC4394973

[9] Smith MH Islam NM Bhattacharyya I Cohen DM Fitzpatrick SG. STAT6 reliably distinguishes solitary fibrous tumors from myofibromas. Head Neck Pathol. (2018) 12:110–7. 10.1007/s12105-017-0836-8 28689369 PMC5873490

[10] Galioto S Valentini V Fatone FMG Rabagliati M Autelitano L Iannetti G. Solitary fibrous tumours of the infratemporal fossa. Two case reports. J Cranio-Maxillofac Surg. (2006) 34(8):494–501. 10.1016/j.jcms.2006.07.864 17157516

[11] Marti-Flich L Schlund M Dapke S Politis C Aubert S Wojcik T. Surgical treatment outcomes of solitary fibrous tumors in the head and neck: a retrospective study. J Cranio-Maxillofac Surg. (2023) 51(6):381–6. 10.1016/j.jcms.2023.05.013 37263831

[12] Lee JH Jeon SH Park CK Park SH Yoon HI Chang JH. The role of postoperative radiotherapy in intracranial solitary fibrous tumor/hemangiopericytoma: a multi-institutional retrospective study (KROG 18-11). Cancer Res Treat. (2022) 54(1):65–74. 10.4143/crt.2021.142 33781051 PMC8756112

[13] Kinslow CJ Rae AI Kumar P McKhann GM Sisti MB Bruce JN. Risk stratification for management of solitary fibrous tumor/hemangiopericytoma of the central nervous system. Cancers (Basel). (2023) 15(3):876. 10.3390/cancers15030876 36765837 PMC9913704

[14] Wu Y Zhao T Cai Y Zheng M Zhang Y Qu Y. Clinical outcomes of solitary fibrous tumors and hemangiopericytomas and risk factors related to recurrence and survival based on the 2021 WHO classification of central nervous system tumors. J Neurosurg. (2024) 140(1):69–79. 10.3171/2023.4.JNS23147 37310060

[15] Na MK Choi KS Lim TH Shin H Lee J Lee H. A systematic review and meta-analysis on the efficacy of postoperative radiotherapy after gross total resection of intracranial solitary fibrous tumors. Sci Rep. (2025) 15:23368. 10.1038/s41598-025-02170-0 40603922 PMC12222894

[16] Chung HR Tam K Han AY Obeidin F Nakasaki M Chhetri DK. Solitary fibrous tumors of the head and neck: a single-institution study of 52 patients. OTO Open. (2022) 6(3):2473974X–221098709. 10.1177/2473974X221098709 PMC9280824 35845143

[17] Ratneswaren T Hogg FRA Gallagher MJ Ashkan K. Surveillance for metastatic hemangiopericytoma-solitary fibrous tumors-systematic literature review on incidence, predictors and diagnosis of extra-cranial disease. J Neuro-Oncol. (2018) 138:447–67. 10.1007/s11060-018-2836-2 29551003

[18] Matthijs V Beckers R Vanden Broecke C Dedeurwaerdere F Van Dorpe J Vanhauwaert D. Central nervous system solitary fibrous tumors: case series in accordance with the WHO 2021 reclassification. Framework for patient surveillance. Acta Neurochir. (2024) 166:414. 10.1007/s00701-024-06304-7 39417883

[19] Gubian A Ganau M Cebula H Scibilia A Todeschi J Santin MN. Intracranial solitary fibrous tumors: a heterogeneous entity with an uncertain clinical behavior. World Neurosurg. (2019) 126:e48–56. 10.1016/j.wneu.2019.01.142 30716501

[20] Baněčková M Martínek P Skálová A Mezencev R Hadravský L Michal M. Solitary fibrous tumors of the head and neck region revisited: a single-institution study of 20 cases and review of the literature. Hum Pathol. (2020) 99:1–12. 10.1016/j.humpath.2020.03.007 32217090

